# An efficient full-length cDNA amplification strategy based on bioinformatics technology and multiplexed PCR methods

**DOI:** 10.1038/srep19420

**Published:** 2016-01-13

**Authors:** Nan Chen, Wei-Min Wang, Huan-Ling Wang

**Affiliations:** 1 Key Lab of Freshwater Animal Breeding, Key Laboratory of Agricultural Animal Genetics, Breeding and Reproduction, Ministry of Education, College of Fishery, Huazhong Agricultural University, 430070, Wuhan, PR China; 2 Freshwater Aquaculture Collaborative Innovation Center of Hubei Province, 430070, Wuhan, PR China

## Abstract

A novel strategy for amplification full-length cDNA and promoter sequences has been developed using bioinformatics technology and multiplexed PCR methods in this study. The amplification of 3′ ends of cDNA is performed according to the modified classic 3′ RACE techniques, therein the more efficient and effective oligo(dT)-anchor primer with hairpin structure is specially designed. For the amplification of 5′ ends of cDNA, two or three-round TAIL-PCR or touch-down PCR using arbitrary degenerate (AD) and sequence-specific reverse (SPR) primers is performed until the 5′ sequence of multi-assembled fragment reaches the exon1 region identified by aligning this fragment to reference genome database. Then another TAIL-PCR or touch-down PCR using genomic DNA as template is conducted to obtain the remaining 5′ and promoter sequences. The 5′ end sites of cDNA are predicted by aligning finally assembled fragment to homologous reference genes of other species, and screening the relative locations of common characteristic *cis*-elements *in silico* on promoter. The putative 5′ ends are further validated by primers corresponding to these predicted sites in cDNAs. This method is suitable for researchers to isolate limited full-length cDNA sequences due to its operability, inexpensiveness, efficiency and speediness.

The transcriptomes of increasing number of species have been sequenced by next generation sequencing (NGS) technique. However, the transcriptomes are always incomplete in length, especially the ends of genes, while the complete genome sequencing is only conducted in some limited species due to the complexity and high cost. Although the NGS technique is produced and developed in the 21^st^ century, researchers still use the PCR technique known as rapid amplification of cDNA ends (RACE) to obtain the full-length cDNA sequences^1^.

In classic RACE, an anchor-sequence tagged oligo(dT) primer is used to reverse mRNA into first-strand cDNA, then the 3′ end sequences of mRNAs are obtained by nest-PCR by the 3′ sequence-specific forward primers together with Outer-R and Inner-R primers (corresponding to the anchor sequence). While a poly(A) tail is tailed to the 3′ ends of first-strand cDNA by terminal transferase and then reversed to second-strand cDNAs by anchor-sequence tagged oligo(dT) primers described above. The Outer-R and Inner-R primers are again used with the 5′ sequence-specific reverse primers (SPRs) to obtain the 5′ ends of cDNA^1^,^2^. This method is complex in operation and low in productivity.

Nowadays, several RACE methods including new RACE and Cap-switching RACE have been developed, especially the 5′ RACE for the difficulty in operation^3^,^4^,^5^,^6^. In new RACE, an anchored RNA adaptor is ligated to the 5′ ends of mRNA before conducting the reverse transcription (RT) reaction. The full-length mRNAs have methylated ‘G’ caps at their termini, when treated with shrimp alkaline phosphatase (SAP) to remove the phosphate, and then T4 RNA ligase is available to ligate the anchor RNA adaptor to the 5′ ends. However, when the mRNAs are incompletely transcribed for lacking the 5′ end, the above progresses are not performed; therefore the full-length cDNAs are only amplified using these anchored and SPR primers^7^,^8^. The method has been applied and developed in cRACE and RLM-RACE^4^,^9^.

The Cap-switching RACE (Cap finder) is performed by moloney murine leukemia virus (MMLV) reverse transcriptase to add extra 2-4 cytosines to the 3′ ends of newly synthesized first-strand cDNAs after reaching the cap structure of the 5′ end of mRNAs^5^,^6^. Then an anchor primer with multiple guanine residues (poly(G)) is added to the reaction mixture and primarily annealed to the exposed poly(C) in the 3′ ends of first-strand cDNA, introducing an adaptor to the first-strand cDNA terminus. Since the cytosine addition is cap-dependent, the anchor primer is only appended to the 3′ ends of full-length cDNA. Actually, the poly(G) primer can also bind to other C-rich sequences of cDNA. The method has further been developed into inverse PCR, T-RACE and step-out PCR^10^,^11^,^12^. Additionally, this method has also been widely applied for the full-length RNA sequencing due to its relatively simple operation and high productivity^13^,^14^,^15^,^16^.

Up to now, some commercial RACE kits from TaKaRa (Full RACE Kit; Full RACE Core Set), Ambion (First Choice RLM-RCAE kit) based on the above new RACE, and Clontech (SMART; SuperSMART; SMARTer) based on the Cap-switching RACE method have been developed. These commercial RACE kits are often tended towards the construction of universal pools of all full-length cDNAs. However, most of the researchers only use this pre-made pool of reverse-transcribed cDNAs to obtain several interested full-length genes. Moreover, the proficiency of researchers in operation is also one of the key success factors because of high-quality RNAs and complex pre-treatment procedures for the RACE technology, and the high price for the commercial RACE kits.

In the present study, we introduce a few, very simple modifications to the cDNA synthesis process for researchers to obtain full-length cDNA sequences. The 3′ ends are amplified by nest-PCR through sequence-specific and anchor primers, while the 5′ ends are obtained by TAIL-PCR or touch-down PCR through SPR and AD primers.

## Results and Discussion

**Figure 1:**
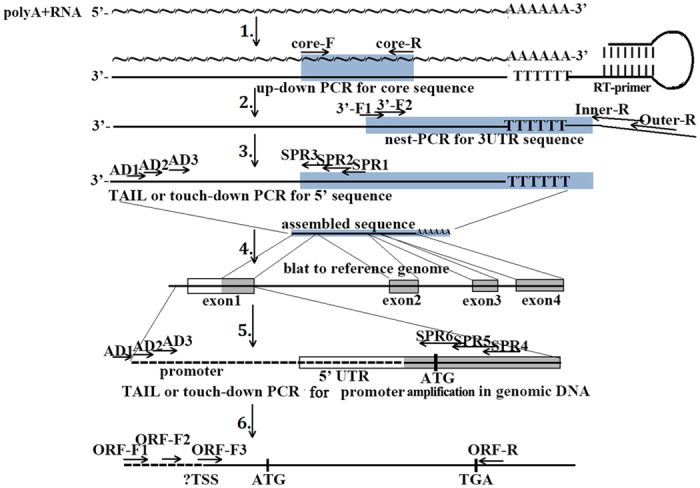
Schematic outline of the 3′ and 5′ RACE approaches. First-strand cDNAs are synthesized using a special hairpin structure oligo(dT)-anchor primer, and the core sequences of target gene are amplified by up-down PCR (step 1). Sequence-specific forward primers are designed together with Outer-R and Inner-R primers to amplify the 3′ end sequences of cDNA through nest-PCR (step 2). Sequence-specific reverse primers (SPRs) referring to the assembled fragments of 3′ end and core sequences are designed and combined with arbitrary degenerate primers (ADs) to amplify 5′ sequences of cDNA by TAIL or touch-down PCR (step 3). After re-assembling, the fragments together with homologous reference gene sequences are aligned to reference genome database for exon distribution analyses (step 4). The PCRs in cDNA will not continue until the multi-assembled fragments reach the exon1 region, then other SPRs referring to the sequence located in exon1 are designed, and one more TAIL or touch-down PCR in genomic DNA are conducted to obtain the promoter sequences (step 5). The 5′ end sites of finally assembled sequence are *in silico* predicted and validated in cDNAs (step 6).

**Figure 2:**
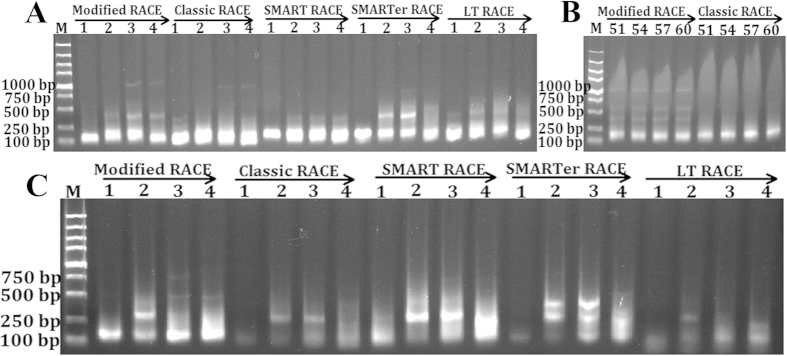
Amplification 3′ end sequences of *M. amblycephala* heat shock transcription factor 2 (*HSF2*) and *PHD3* in cDNAs reversed by oligo(dT) primers from modified RACE, classic RACE, SMART RACE, SMARTer RACE and life technologies (LT) Gene Race Kit, respectively. The 3′ end sequence of *HSF2* is amplified in four cDNA templates and only templates reversed by modified and classic RT primers achieve the correct bands (**A**). Then *HSF2* 3′ end is further amplified in different temperature conditions (51, 54, 57 and 60 °C), and cDNA templates reversed by hairpin structure RT primer performes better (**B**). These templates are also used to amplifiy 3′ end of *PHD3* in one-round PCR, and cDNA templates from modified RACE and SMART can obtain weak target bands (**C**).

**Figure 3:**
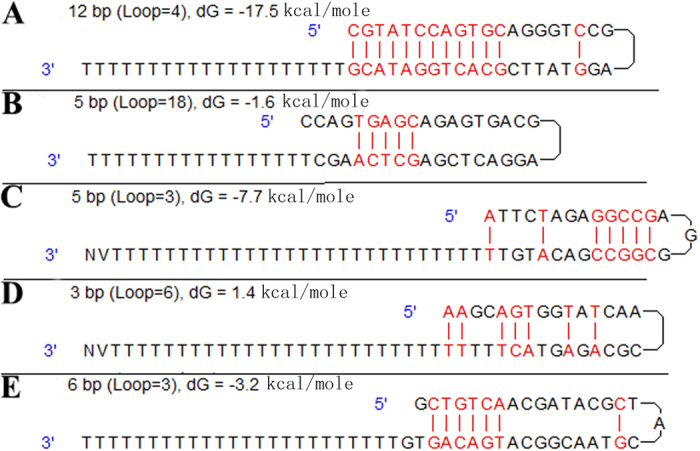
The predicted secondary structures of RT-primer sequences. RT-primers are coming from modified RACE (**A**), classic RACE (**B**), SMART RACE (**C**), SMARTer RACE (**D**) and life technologies Gene Race Kit (**E**), respectively. RT-primers from modified and SMART RACE are likely to form hairpin structure during the RT reactions, and only oligo(dT) sequence is exposed, as a result to enhance the RT reaction specificity.

We report an efficient strategy for acquiring the full-length cDNAs by multiplexed PCR combined with bioinformatics analysis (Fig. 1). Using this approach, we have identified the full-length cDNA and the promoter sequences of *Megalobrama amblycephala PHD* family, *Litopenaeus vannamei HSP70* and *Ctenopharyngodon idella EDN1*, respectively. The developments of all the RACE techniques are focused on the 5′ RACE, since it is difficult to obtain the complete 5′ ends of mRNA from conventional cDNA libraries, while the 3′ RACE development is neglected for its simple operation. In the present study, the oligo(dT)-anchor primer with a special hairpin structure was developed, and the efficiency and specificity were compared by amplifying the 3′ ends of low expressed heat shock transcription factor 2 (*HSF2*) and high expressed *PHD3* genes of *M. amblycephala* cDNAs reversed by RT primers form this study, classic 3′ RACE, SMART RACE, SMARTer RACE and life technologies (LT) Gene Race Kit, respectively. The results revealed that the 3′ end of *HSF2* was only obtained in cDNAs reversed by hairpin structure and classic 3′ RACE RT primers, and cDNAs reversed by hairpin structure RT primer showed more clear bands than classic 3′ RACE RT primer in a wider range of denaturation temperature (Fig. 2A,B and Supplementary Figure S1, S2). Additionally, only the first-round PCR in *PHD3* 3′ end amplification indicated that cDNAs reversed by hairpin structure and SMART 3′ RACE RT primers showed the weak and correct bands (Fig. 2C and Supplementary Figure S3). All these results indicating that the oligo(dT)-anchor primer in this study showed higher efficiency and specificity, and further analysis was suggested that the oligo(dT)-anchor primers from our study and SMART primers more easily formed the hairpin structure during RT reactions (Fig. 3).

**Figure 4:**
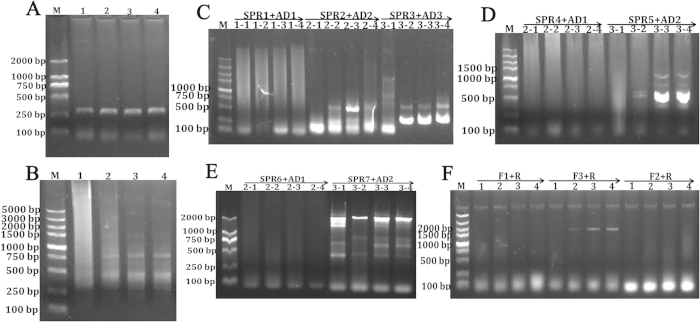
Amplification of the full-length cDNA and promoter sequences of *M. amblycephala PHD1*. The core sequence of *PHD1* is obtained from EST database and validated (**A**). Then sequence-specific forward primers are designed together with Outer-R and Inner-R primers to amplify the 3′ end sequence by nest-PCR (**B**). After 3′ end and core sequences assembling, SPRs are designed together with ADs to amplify the 5′ sequence by three-round TAIL-PCR (**C**). Since the re-assembled sequence can not reach the exon1 region after aligning, another step two-round TAIL-PCR is conducted to obtain extra 5′ unknown sequence (**D**). Then the third-assembling sequence is aligned again into zebrafish genome database to analyse the exon distributions, and another SPRs located in the exon1 region are designed together with ADs to amplify the promoter sequence by TAIL-PCR in genomic DNA (**E**). Through *in silico* analysis, the predicted 5′ end sites are identified and validated in cDNAs using ORF-F1, ORF-F2, ORF-F3 and ORF-R primers (**F**).

**Figure 5:**
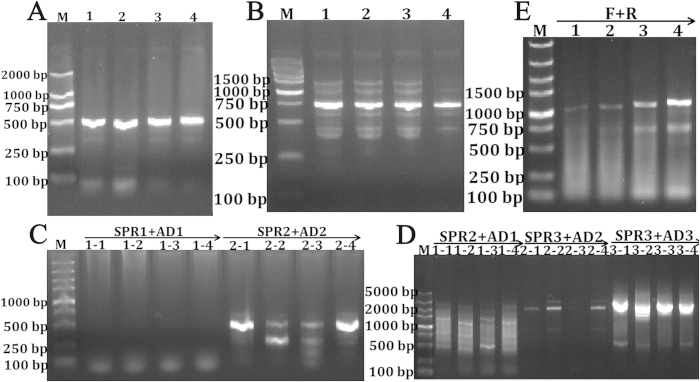
Amplification of the full-length cDNA and promoter sequences of *M. amblycephala PHD3*. The core sequence of *PHD3* is obtained from EST database and validated (**A**). Then sequence-specific forward primers are designed together with Outer-R and Inner-R primers to amplify the 3′ end sequence by nest-PCR (**B**). After 3′′ end and core sequences assembling, SPRs are designed together with ADs to amplify the 5′ sequence by two-round TAIL-PCR (**C**). Then the re-assembled sequence is aligned to analyse the exon distributions, and another SPRs located in exon1 region are designed together with ADs to amplify promoter sequence by three-round TAIL-PCR in genomic DNA (**D**). Through *in silico* analysis, the predicted 5′ end site is identified and validated in cDNAs using ORF-F and ORF-R primers (**E**).

The core sequences of *PHD* family in *M. amblycephala* are obtained by transcriptome database and for those species that have EST sequences will significantly reduce the time of RACE experiments. With the development of NGS and other sequencing techniques, transcriptomes of hundreds of species have been reported nowadays. However, conducting 5′ RACE to obtain the complete sequences of these ESTs is challenging, especially for isolation of some longer sequences, and researchers have to give attempts by the optimization of various amplification conditions, therefore, several 5′ RACE techniques have been developed. In this study, since the cDNA sequence of *PHD1* (2672 bp, GenBank Accession: KT428344, *PHD1* promoter GenBank Accession: KT428342) was longer, the 5′ end sequence was obtained by two steps TAIL-PCR (Fig. 4 and Supplementary Figure S4), relatively *PHD3* (1622 bp, GenBank Accession: KT428345, *PHD3* promoter GenBank Accession: KT428343) was shorter (Fig. 5 and Supplementary Figure S5). Although it needs more time to complete, the step to step overlapping extension will guarantee to get the target sequence.

In *PHDs* 5′ end sequence amplification, TAIL-PCR is primarily used and other attempts are also useful, for example using the first-round TAIL-PCR products to conduct second-round touch-down PCR, using other more effective arbitrary degenerate (AD) primers in different experiments. In *PHD3* 5′ end sequence amplification, the second-round TAIL-PCR products were detected by gel electrophoresis and the correct products were quite clear with no need to perform the third-round PCR (Fig. 5 and Supplementary Figure S5), which saved the experiment time.

**Figure 6:**
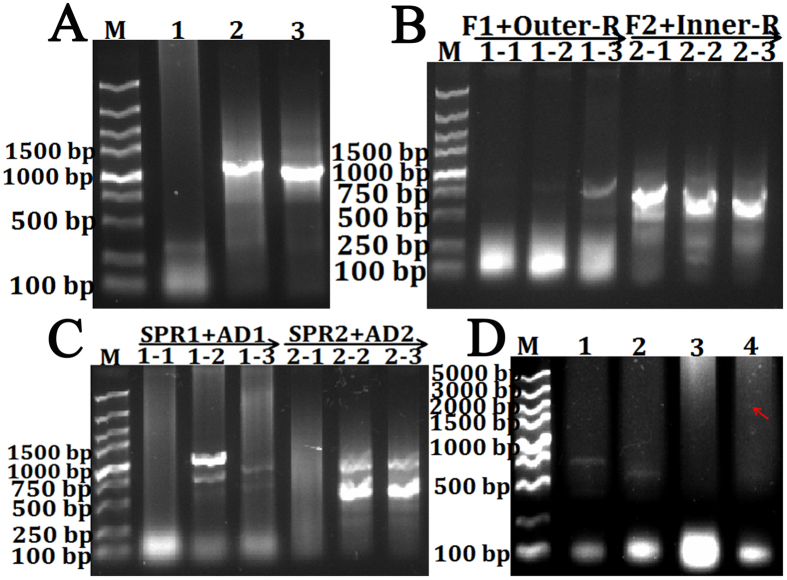
Amplification of the full-length cDNA and promoter sequences of *L. vannamei HSP70*. The core sequence of *HSP70* is amplified by degenerate primers through touch-down PCR (**A**). Then sequence-specific forward primers are designed together with Outer-R and Inner-R primers to obtaine the 3′ end sequence by nest-PCR (**B**). *In silico* analysis indicats that *HSP70* has only one exon, thus SPRs and ADs are used to amplify 5′ and promoter sequence through two-round touch-down PCR in genomic DNA (**C**). Through *in silico* analysis, the predicted 5′end site is identified and validated in cDNAs using ORF-F and ORF-R primers (**D**).

Generally, amino acid sequences are more conserved among species, therefore the core sequence of *L. vannamei HSP70* is generated using the primer designed by codeHop according to amino acid homology, because *L. vannamei* transcriptome and genome data of related species are lacked. In addition, genome structure including exon numbers and the distributions are usually conservative during evolution, so it will be easy to identify the exon distributions and confirm the promoter regions. Bioinformatic analysis showed that *HSP70* was strictly conserved and only had one exon in genomes of all studied organisms, therefore we easily and quickly isolated the full-length cDNA and promoter sequences of *L. vannamei HSP70* by touch-down PCR (Fig. 6 and Supplementary Figure S6), and demonstrated that this method was accurate and efficient by sequence alignment with partial sequence of the previously published *L. vannamei HSP70*.

**Figure 7:**
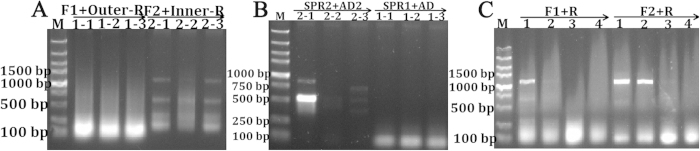
Amplification of the full-length cDNA and promoter sequences of *C. idella EDN1*. The core and 3′ end sequence of *EDN1* is obtained by modified degenerate primers through touch-down PCR (**A**). Since introns in length of zebrafish *EDN1* are short, the 5′ end and promoter sequences of *C. idella EDN1* are all amplified in genomic DNA (**B**). *In silico* analysis is used to identify the *EDN1* exons and 5′ end sites, and then validated by ORF-F1, ORF-F2 and ORF-R primers in cDNAs (**C**).

The *C. idella EDN1* gene is lowly expressed and not conserved among different species, and one conserved amino acid region is only presented in codeHop. Therefore, two forward primers in this region were designed and modified to decrease denaturation temperature of degenerate primers in order to obtain the core and 3′ end sequences by combining the Outer-R and Inner-R primers (Fig. 7A and Supplementary Figure S7). Since introns in length of zebrafish *EDN1* were short, the remaining 5′ and promoter sequences of *C. idella EDN1* were all amplified in genomic DNA (Fig. 7B and Supplementary Figure S7), then zebrafish EDN1 amino acid sequence (GenBank Accession: NP_571594.1) was aligned to this genomic sequence by online NCBI tBLASTn program to identify *C. idella EDN1* exon sequences, and then validated by ORF amplification (Fig. 7C and Supplementary Figure S7).

The method described herein offers several advantages over other protocols currently used. The operations in RT reaction and 5′ amplification are quite easy, quick and inexpensive. The step to step overlapping extension is quite suitable for long gene cloning. For those genes without transcriptome data, this method could be used to obtain the core sequence efficiently. Now this technique has been successfully applied in isolation of the full-length and promoter sequences of several genes.

## Materials and Methods

### Total RNA and genomic DNA extraction

Total RNA was extracted from liver tissues of *M. amblycephala*, *L. vannamei* and *C. idella* by TRIzol reagent (Life Technologies, USA) according to the manufacturer′s protocol. Genomic DNA was extracted from fish fin clip, shrimp muscle using the traditional phenol-chloroform method. DNA and RNA concentrations were measured using the NanoDrop 2000 (Thermo Fisher Scientific, USA). All investigations were conducted in accordance with the ethical standards and according to the national and international guidelines and have been approved by College of Fishery Ethics Committee of Key Lab of Freshwater Animal Breeding.

### First-strand cDNA synthesis

First-strand cDNA synthesis was performed by reverse transcriptase kit (Promega, USA) as follows: 2 μg of total RNA and 50 μmol oligo(dT)-anchor primer (Table 1) were incubated at 70 °C for 5 min and then quickly chilled on ice before addition of 2 μL of 10 mM dNTPs, 5 μL of 5x buffer, 1 μL of 40 U RNAase inhibitor and 1 μL of 200 U M-MLV reverse transcriptase in a total of 25 μL mixture, then the mixture was incubated at 42 °C for 1 h.

### PCR primers

The primers were all designed using the online primer designing tool in NCBI (http://www.ncbi.nlm.nih.gov/tools/primer-blast/) and shown in Table 1. Among these primers, three arbitrary degenerate primers (AD1, AD2 and AD3) were synthesized as previously described^17^.

### Isolation of core sequences of genes

The core sequences of *M. amblycephala* prolyl-hydroxylase 1 (*PHD1*) and 3 (*PHD3*) were isolated through the published *M. amblycephala* transcriptome^18^ by local tBLASTn program (Version: 2.2.30) from NCBI. *L. vannamei* heat shock protein 70 (*HSP70*) and *C. idella* endothelin 1 (*EDN1*) core sequences were amplified by degenerate primers (Table 1) designed by online tool codeHop (http://blocks.fhcrc.org/codehop.html) with default parameters except that zebrafish genetic code was selected as standard encoded mode^19^.

### Nest-PCR for amplification of the 3′ end sequences

Usually 1 or 2 sequence-specific forward primers were designed according to the core sequences, together with Outer-R and Inner-R primers (Table 1) to perform PCR in cDNAs. The first-round PCR products were 50-fold diluted as the template for the second-round PCR.

### TAIL-PCR or touch-down PCR for amplification of the 5′ sequences

The core sequence and the 3′ end sequence were assembled by DNAStar software (Version: 7.1.0) after identification. For amplification of the 5′ sequences, TAIL-PCR and touch-down PCR (Table 2 and Table 3) were carried out using 2 or 3 long sequence-specific reverse primers (SPR1, SPR2 and SPR3) based on the 5′ terminus region of assembled fragments. Briefly, SPR1 and AD1 were used to conduct first-round PCR by TAIL-PCR or touch-down PCR, and then the products were 50-fold diluted as the template for second-round PCR. The primer pairs, SPR2/AD2, and SPR3/AD3 were considered in the second-round and third-round TAIL-PCR or touch-down PCR, respectively. Finally, the third-round PCR products were identified by sequencing and re-assembled.

### Promoter amplification

In cDNA amplification, the 5′ ends of gene can not be obtained by the above methods, thus the genomic DNA is used to isolate the 5′ end sequences. In fish, the model species zebrafish genome and reference gene sequences were available. The above assembled sequences and the corresponding zebrafish reference genes were BLAT in zebrafish genome database (danRer10, http://genome.ucsc.edu/). The assembled sequence locations in zebrafish genome were compared with the reference gene locations to obtain the exon distributions. Therefore, the 5′ ends of gene will be obtained by amplification of promoter sequences for TAIL-PCR or touch-down PCR in genomic DNA using another 2 or 3 SPR primers located in exon1, when the above assembled sequences reached the exon1 region.

### Transcription start site (TSS) identification

After promoter amplification, two steps were used to identify whether the amplified genomic sequence was the accurate core promoter of intended genes according to the below bioinformatics procedures. In fish species, firstly, the genomic sequence was aligned to zebrafish genome database again to check the zebrafish gene name and sequence in genomic sequence matched region. Secondly, the putative core promoter sequence was up-loaded to online JASPAR CORE Vertebrata database (http://jaspar.genereg.net/) with default parameters to scan the core promoter specific *cis*-elements, such as TATA box, CAAT box, GAGA box, etc^20^. For a special gene, there were specific transcription factor binding sites (TFBS) on the core promoters.

After promoter identification, the promoter and target gene cDNA sequences were assembled again to obtain finally assembled fragments. The sequences alignment by BLAST was performed between the finally assembled fragments and homologous reference genes from other species to find the potential 5′ end sites. Meanwhile, the basal core promoter region could be roughly identified based on core promoter specific *cis*-elements locations. Integrally, several putative transcription start sites (TSSs) were predicted and further identified by designing forward primers near the putative TSS regions and the reverse primers near the assembled cDNA translation termination codon regions. Finally, coding sequences were also validated using cDNA as templates.

## Additional Information

**How to cite this article**: Chen, N. *et al.* An efficient full-length cDNA amplification strategy based on bioinformatics technology and multiplexed PCR methods. *Sci. Rep.* **6**, 19420; doi: 10.1038/srep19420 (2016).

## Supplementary Material

Supplementary Information

## Figures and Tables

**Table 1:** Primers used in this study.

Primer names	Primer sequences (5′-3′)	Applications
PHD1-core	F: TTACCCTGGCAACGGAACAG	Amplification core sequence of PHD1, 301 bp
	R: CTTCTCCTTAGCTTCGGCCC	
PHD3-core	F: TCTTTCTGTGAGGTGGCTGTC	Amplification core sequence of PHD3, 465 bp
	R: ATGGGTTAACGGGACCGAGA	
HSP70-core	F: GTGTTCCAGCATGGCAAGGTGGARATCMTCGCC	Amplification core sequence of HSP70, 1320 bp
	R: ACGTTCAGGATGCCGTTGGCGTCRATGTCGA	
EDN1-core	F1: CCTGGATAAAGAGTGCGTCTACTTYTGYCA	Amplification core and 3′ end sequences of EDN1, 1139 bp
	F2: GCGAACGGTGTCATATGGAYTTGGAAAYGC	
Hairpin structure RT primer	CGTATCCAGTGCAGGGTCCGAGGTATTCGCACTGGATACGT20	Reverse mRNA into cDNA
Outer-R	CAGTGCAGGGTCCGAGGTAT	Amplification 3′ ends of cDNA
Inner-R	CCGAGGTATTCGCACTGGATACGTTT	Amplification 3′ ends of cDNA
Classic RT primer	CCAGTGAGCAGAGTGACGAGGACTCGAGCTCAAGCT17	Reverse mRNA into cDNA
Classic-Outer	CCAGTGAGCAGAGTGACG	Amplification 3′ ends of cDNA
Classic-Inner	GAGGACTCGAGCTCAAGC	Amplification 3′ ends of cDNA
SMART RT primer	ATTCTAGAGGCCGAGGCGGCCGACATGT30VN	Reverse mRNA into cDNA
SMART-R	ATTCTAGAGGCCGAGGCGGCCGACATG	Amplification 3′ ends of cDNA
SMARTer RT primer	AAGCAGTGGTATCAACGCAGAGTACT30VN	Reverse mRNA into cDNA
SMARTer-R	AAGCAGTGGTATCAACGCAGAGT	Amplification 3′ ends of cDNA
LT RT primer	GCTGTCAACGATACGCTACGTAACGGCATGACAGTGT24	Reverse mRNA into cDNA
LT-R	GCTGTCAACGATACGCTACGTAACG	Amplification 3′ ends of cDNA
HSF2-3	F: TAGAGTTGATGGACTATCTGGACAGTATTGA	Amplification 3′ end of HSF2, 1060 bp
PHD1-3	F: TAACCTGCATCTACTATCTGAACAAGGA	Amplification 3′ end of PHD1, 1291 bp
PHD3-3	F: TTTCTTATTAACACTCATAGACAAACTCATTTC	Amplification 3′ end of PHD3, 770 bp
HSP70-3	F1: TCTCATCAAGCGTAACACCACCAT	Amplification 3′ end of HSP70, 881 bp
	F2: GACTCAGACCTTCACCACCTACTC	
AD	-1: NTCGASTWTSGWGTT	Amplification promoter and 5′ sequences
	-2: NGTCGASWGANAWGAA	
	-3: WGTGNAGWANCANAGA	
PHD1-5	SPR1: GAAAATGAGCAGACGGTCAAAGAGAG	Amplification promoter and 5′ sequence of PHD1
	SPR2: GTGGATCTGCAATAAACCTCCATGAAC	
	SPR3: CATATCCTGTTCCGTTGCCAGGGTAA	
	SPR4: CGGATGTTCTTAGAGGGGATGCTCTTTTGGATG	
	SPR5: CCCGCTGCGATTTAGGGTCTCCACTTCCTCCAG	
	SPR6: CCATAGAACTTCATACAGGGAACTATGTACTG	
	SPR7: GTGTGCTTTTACTAGAGTCTGTTTTAGG	
PHD3-5	SPR1: TCTTTCTGTGAGGTGGCTGTCAGATCTCTA	Amplification promoter and 5′ sequence of PHD3
	SPR2: TGACCTTGCATGAATGCTTTTGCCCAGTTGACC	
	SPR3: TGAGTGTTAATAAGAAATTGATAGCCTCCGTGC	
	SPR4: ATGTTATTTTATCCCCTCTGATGTTTGTCCTGC	
HSP70-5	SPR1: CTTCATTTTGATGAGCACCATCGAG	Amplification promoter and 5′ sequence of HSP70
	SPR2: GTAGAAGGTCTTCTTGTCTCCCTTG	
EDN1-5	SPR1: GTTTTGTCTTTGCTATCTGCACATTT	Amplification promoter and 5′ sequence of EDN1
	SPR2: ATTTCCAAATCCATATGACACCGTTC	
PHD1-ORF	F1: TGCTTCAAGACCATCGTAAATTAATATTAATGAG	Identification 5′ end of PHD1 and validation its ORF, 1824 bp
	F2: CAGTGTTATATCAGATACGAAGCCTACT	
	F3: CCTTAATCTGGAAATAAACAGCCATGGAAAATAG	
	R: AAGCAATTCAGACTTGTTGGATGTTTGAGG	
PHD3-ORF	F: TCACTTTAAAAAAACTTTAGCTGATTTGA	Identification 5′ end of PHD3 and validation its ORF, 832 bp
	R: GCTAAGAAATAAGATGATAGACGCA	
HSP70-ORF	F: GTGGTTGGGTCTTCATGATAATGCTT	Identification 5′ end of HSP70 and validation its ORF, 2131 bp
	R: CCACCAACATCATAAATAAAATGCGG	
EDN1-ORF	F1: GTTTGTGTATTTGATGGTCTATCACCGTCTG	Identification 5′ end of EDN1 and validation its ORF, 1216 bp
	F2: GTCTGAGTCACATCCGCGTCTTGCATAAG	
	R: TTTTACAGTGTATGAAGTCCAATAACAAGA	

**Table 2:** The parameters of TAIL-PCR.

Reactions	Program NO.	Number of cycles	Cycle Parameters
First-round PCR	1	1	95 °C 2 min, 97 °C 1 min
	2	10	95 °C 20 s, 63 °C 25s, 72 °C 1 min
	3	1	95 °C 20 s, 25 °C 1 min, ramping to 72 °C over 1 min, 72 °C 1 min
	4	10	95 °C 20 s, 30 °C 25 s, 72 °C 1 min
	5	12	95 °C 20 s, 63 °C 25 s, 72 °C 45 s
			95 °C 20 s, 63 °C 25 s, 72 °C 45 s
			95 °C 20 s, 30 °C 25 s, 72 °C 45 s
	6	1	72 °C 2 min, 30 °C ∞
Second-round PCR	1	1	95 °C 3 min
	2	12	95 °C 20 s, 60 °C 25 s, 72 °C 45 s
			95 °C 20 s, 60 °C 25 s, 72 °C 45 s
			95 °C 20 s, 48 °C 25 s, 72 °C 45 s
	3	1	72 °C 2 min, 30 °C ∞
Third-round PCR	1	1	95 °C 3 min
	2	12	95 °C 20 s, 60 °C 25 s, 72 °C 45 s
			95 °C 20 s, 60 °C 25 s, 72 °C 45 s
			95 °C 20 s, 48 °C 25 s, 72 °C 45 s
	3	1	72 °C 2 min, 30 °C ∞

**Table 3:** The parameters of touch-down PCR.

Reaction	Program NO.	Number of cycles	Cycle Parameters
Touch-down PCR	1	1	95 °C 3 min,
	2	21	95 °C 20 s, 65 °C to 55 °C 45 s (decrease 0.5 °C each cycle), 72 °C 45 s
	3	20	95 °C 20 s, 55 °C 20 s, 72 °C 45 s
	4	1	72 °C 2 min, 30 °C ∞
