# Supplementary material for: An efficient full-length cDNA amplification strategy based on bioinformatics technology and multiplexed PCR methods
Source: Sci Rep. 2016 Jan 13;5:19420. doi: 10.1038/srep19420 (PMC4725349; doi:10.1038/srep19420)
Supplement: Supplementary Information [file srep19420-s1.doc]

An efficient full-length cDNA amplification strategy based on bioinformatics technology and multiplexed PCR methods

Nan Chen^1^, Wei-Min Wang^1^, Huan-Ling Wang^1, 2 *^

^1^ Key Lab of Freshwater Animal Breeding, Key Laboratory of Agricultural Animal Genetics, Breeding and Reproduction, Ministry of Education, College of Fishery, Huazhong Agricultural University, 430070, Wuhan, PR China

^2^ Freshwater Aquaculture Collaborative Innovation Center of Hubei Province, 430070, Wuhan, PR China

* Corresponding author. Tel.: +86 027 87282113; Fax: +86 027 87282114.

E-mail address: hbauwhl@hotmail.com (Huan-Ling Wang).

All the agarose gel electrophoresis results were the original pictures with DNA Marker.


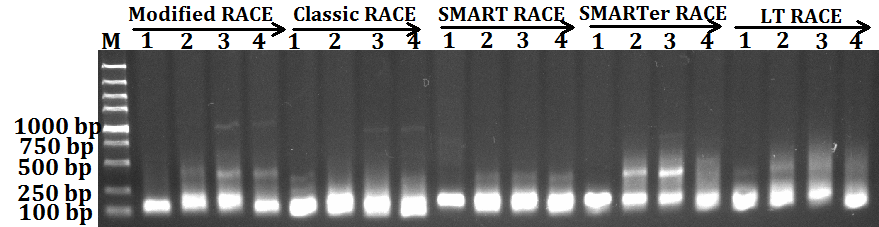


Supplementary figure S1. The 3’ end sequence of *HSF2* is amplified in four cDNA templates and only templates reversed by modified and classic RT primers achieve the correct bands.


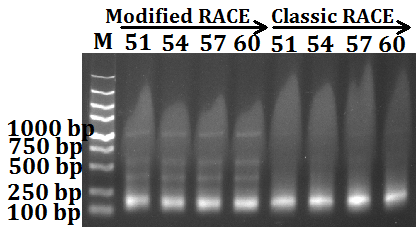


Supplementary figure S2. Then *HSF2* 3’ end is further amplified in different temperature conditions (51, 54, 57 and 60°C).


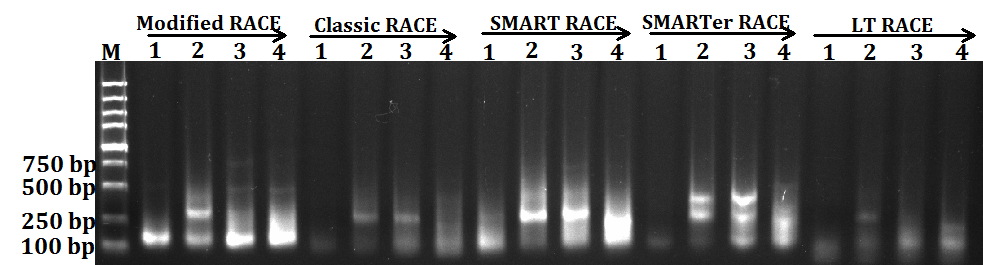


Supplementary figure S3. Amplification 3’ end of *PHD3* in one-round PCR using cDNA reversed by RACE, classic RACE, SMART RACE, SMARTer RACE and life technologies (LT) Gene Race Kit, respectively.


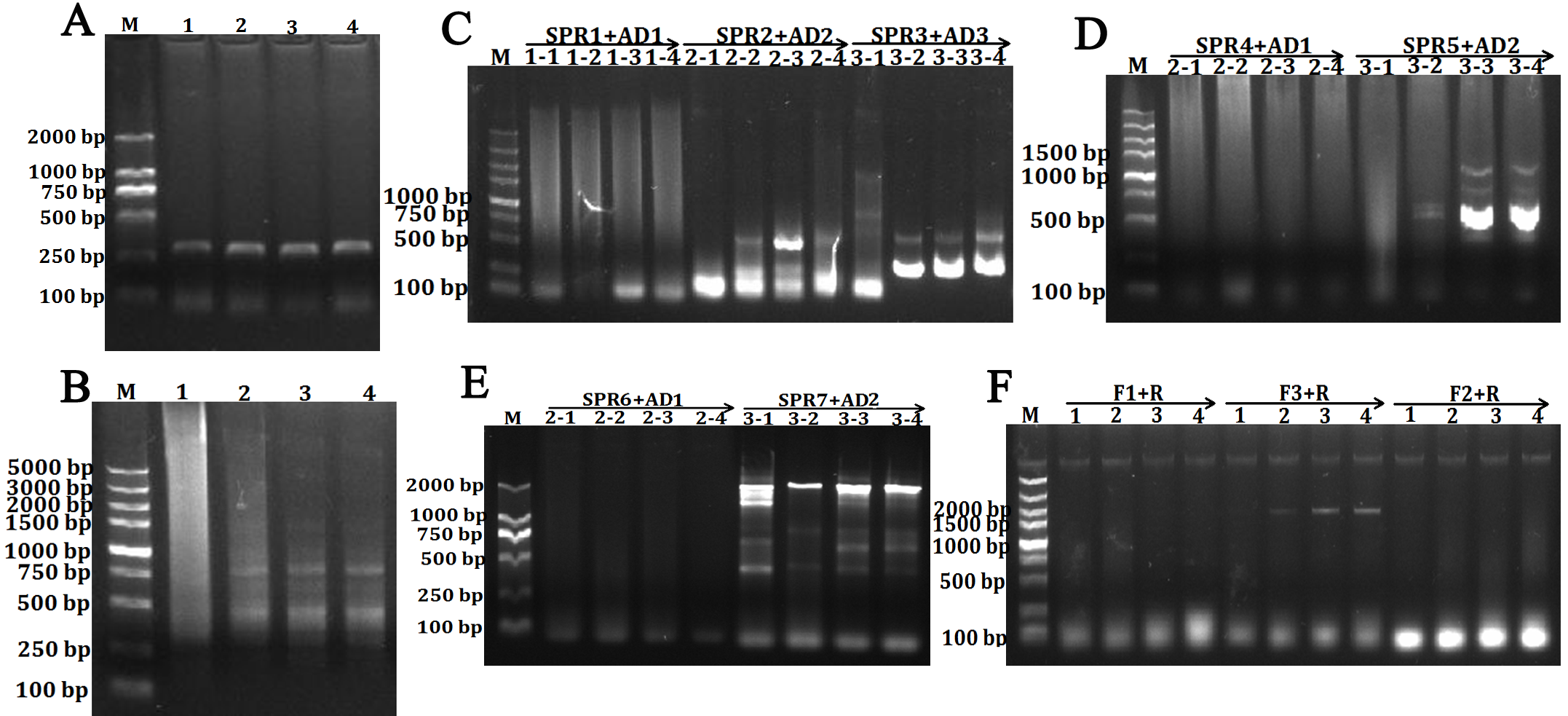


Supplementary figure S4. Amplification of the full-length cDNA and promoter sequences of *M. amblycephala PHD1*.


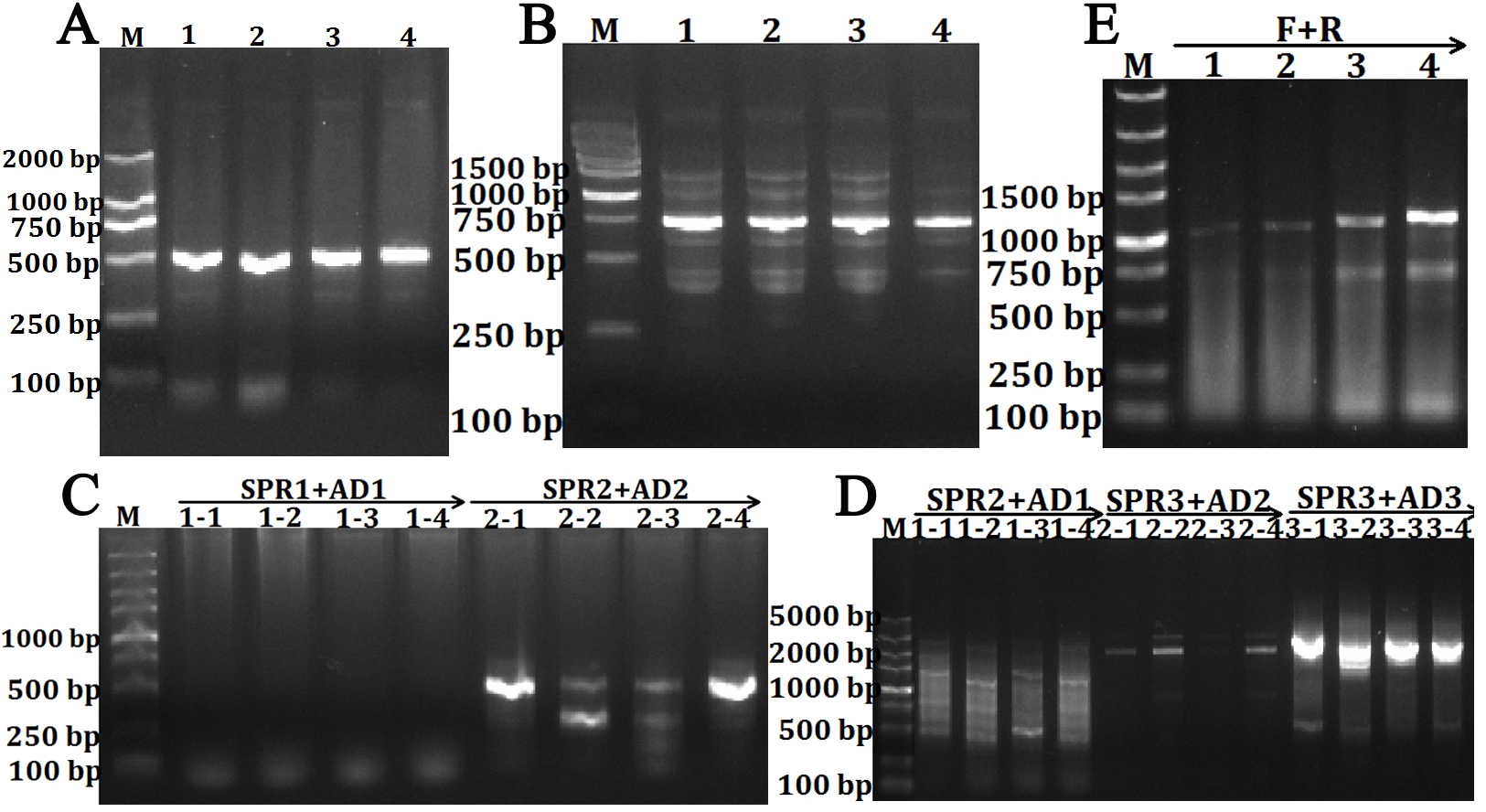


Supplementary figure S5. Amplification of the full-length cDNA and promoter sequences of *M. amblycephala PHD3*.


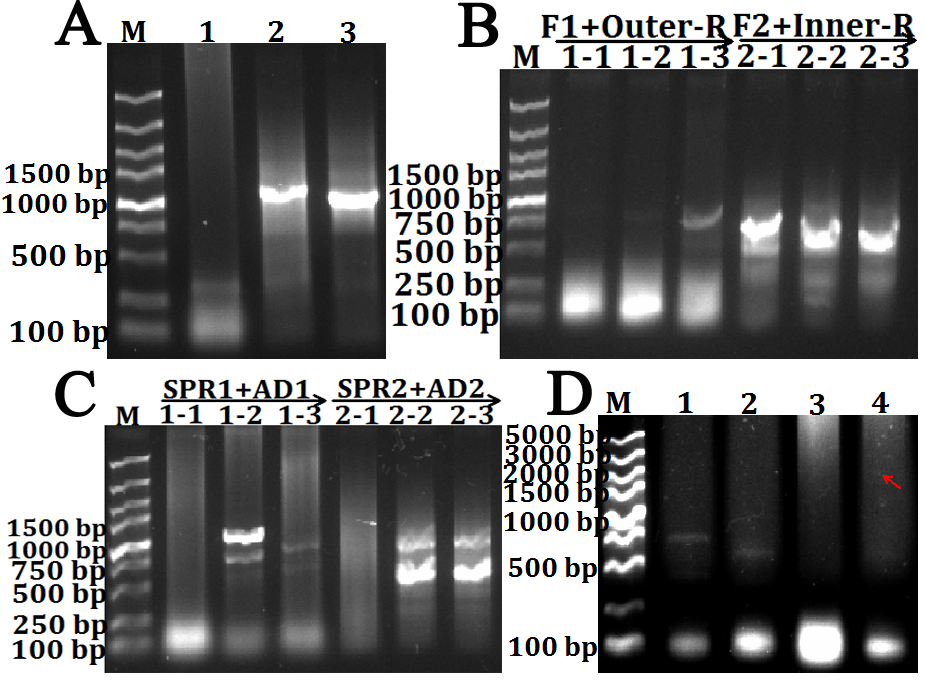


Supplementary figure S6. Amplification of the full-length cDNA and promoter sequences of *L. vannamei HSP70*.


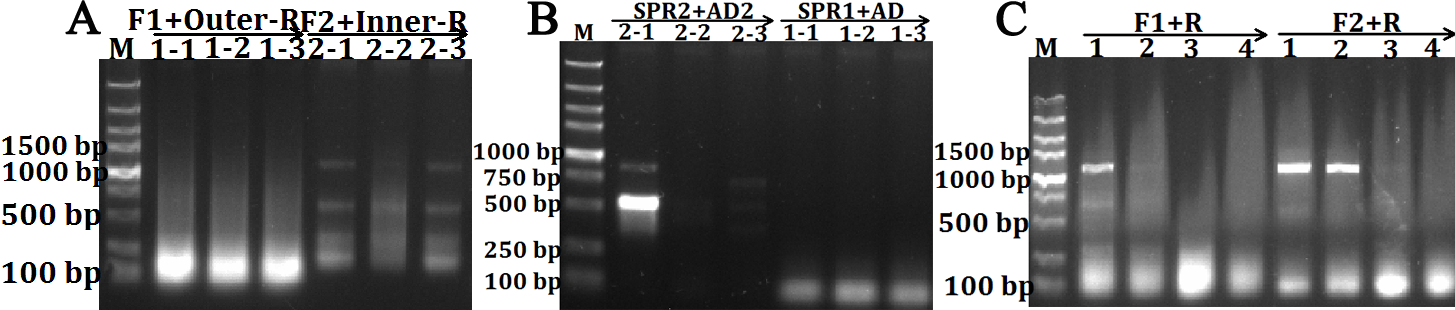


Supplementary figure S7. Amplification of the full-length cDNA and promoter sequences of *C. idella EDN1*.
